# Enhancement Effect of Chitosan Coating on Inhibition of Deoxynivalenol Accumulation by *Litsea cubeba* Essential Oil Emulsion during Malting

**DOI:** 10.3390/foods10123051

**Published:** 2021-12-08

**Authors:** Zhengcong Peng, Wenxu Feng, Guolin Cai, Dianhui Wu, Jian Lu

**Affiliations:** 1 The Key Laboratory of Industrial Biotechnology, Ministry of Education, School of Biotechnology, Jiangnan University, 1800 Lihu Road, Wuxi 214122, China; sd3961471@126.com (Z.P.); fengwenxu0526@163.com (W.F.); glcai@jiangnan.edu.cn (G.C.); wudianhui@jiangnan.edu.cn (D.W.); 2 National Engineering Laboratory for Cereal Fermentation Technology, Jiangnan University, 1800 Lihu Road, Wuxi 214122, China; 3 Jiangsu Provincial Research Center for Bioactive Product Processing Technology, Jiangnan University, 1800 Lihu Road, Wuxi 214122, China; 4 Institute of Food Biotechnology, Jiangnan University, 99 Wanshou Road, Rugao 226500, China

**Keywords:** essential oil, emulsion, deoxynivalenol, malting, antifungal

## Abstract

The purpose of this work was to study the enhancement effect of chitosan coating on inhibition of deoxynivalenol (DON) accumulation by *Litsea cubeba* essential oil emulsion during malting. Firstly, the primary emulsion suitable for malting process was screened and the improvement effect of chitosan coating on the properties of primary emulsion was studied. On this basis, chitosan-based *Litsea cubeba* essential oil emulsion was applied to malting processing. The results showed that the primary emulsion of *Litsea cubeba* essential oil had good antifungal properties and a minimal effect on the germinability of barley compared with other primary emulsions. The addition of chitosan can improve the physical stability and antifungal ability of the emulsion and reduce the effect of the emulsion on barley germination. When 100 g of chitosan-based *Litsea cubeba* essential oil emulsion (40 mg/g) was applied to the malting process, the germination rate of barley was 87.7% and the DON concentration of finished malt was reduced to 690 μg/kg, which was 20.9% lower than that of the control. Meanwhile, the other indexes of malt produced by secondary emulsion treatment (after adding chitosan) increased significantly compared with those of malt produced by primary emulsion. This study was of great significance for the application of emulsion to inhibit the accumulation of mycotoxin during malting.

## 1. Introduction

Deoxynivalenol (DON) is a type B trichothecene mycotoxin, which is produced by *Fusarium* when it infects cereals, fruits, and vegetables [1,2]. Barley malt is the main raw material of beer production, which is prepared by soaking, germinating, and baking under a certain temperature and humidity. There is a risk of DON contamination in the production and storage process of malt. Meanwhile, DON has good thermal stability and water solubility, which is not easy to remove in the beer production process and will eventually remain in the finished beer [3,4,5]. Studies have shown that excessive intake of DON can produce toxic reactions, and long-term low doses can also cause damage to the body [6,7]. Therefore, the residual of DON in beer will pose a threat to the health of consumers.

Controlling the growth of *Fusarium* during malting is an effective strategy to control the production of DON in beer [5]. At present, spraying chemical fungicides is the main method to inhibit the growth and reproduction of grain fungi, but the abuse of chemical fungicides will cause serious environmental pollution [8,9,10]. To this end, researchers have developed several relatively safe alternative methods, such as radiation treatment [11] and the microbial method [12]. However, radiation treatment is difficult to scale, and the introduction of microorganisms may also cause secondary pollution. Therefore, the development of green, efficient, and new antifungal agents is the key to current research.

Essential oils (EOs) are natural fungicides with good broad-spectrum antibacterial activity [13,14]. In addition, essential oils are biodegradable, highly volatile, and low residue, and will not have a negative impact on the ecological environment. However, essential oils are usually insoluble in water, which makes them difficult to use in malting [15]. Fortunately, it was found that the encapsulation of EOs in emulsion can improve their water solubility and stability and emerged obviously better antimicrobial activity than free Eos [16,17,18,19]. For example, cinnamon essential oil nanoemulsion can completely inhibit the radial mycelial growth of *Aspergillus niger*, while free cinnamon essential oil showed only 75% inhibition at the same content [20]. Although the emulsion-based delivery system is one of the viable candidates, it can be challenging to prepare physically stable emulsions [21]. Chitosan is a cationic polysaccharide with broad-spectrum antibacterial effect. Research has shown that chitosan can be used as the wall material of emulsion to improve the stability and enhance the overall antibacterial performance [22,23,24,25]. It is a feasible strategy to combine essential oil and chitosan to establish the antifungal system, but there is no report that chitosan-based emulsion was applied to inhibit the growth of *Fusarium graminearum* during malting.

The aim of our work is to apply chitosan-based emulsion to the preparation of barley malt and study the enhancement effect of chitosan coating on inhibition of deoxynivalenol accumulation by *Litsea cubeba* essential oil emulsion during malting. The research results of our work have broad application prospects in the beer malt industry, and can even be further expanded in grain processing, feed processing, and other industries.

## 2. Materials and Methods

### 2.1. Materials

Lecithin, cinnamon oil, Origanum oil, and chitosan (MW = 30000) were purchased from Shanghai Macklin Biochemical Technology Co., Ltd., (Shanghai, China). *Litsea cubeba* oil, patchouli oil, and medium-chain triglyceride were purchased from Shanghai Yuanye Biotechnology Co., Ltd., (Shanghai, China). Fennel oil and citronella oil were purchased from Sigma Company of Shanghai (Shanghai, China). Clove oil, acetonitrile (chromatographic pure), methanol (chromatographic pure), acetic acid, sodium acetate dihydrate, and other reagents were purchased from Sinopharm Chemical Reagent Co., Ltd., (Shanghai, China). The plant genome extraction kit was purchased from Nanjing Vazyme Biotechnology Co., Ltd., (Nanjing, China). Potatoes and mung beans were purchased from local supermarkets (Wuxi, China), while barley infected with *Fusarium* head blight was provided by Jiangsu Agricultural Reclamation Malt Company (Sheyang, China).

### 2.2. Apparatus

Zeta potential and particle size was measured by a zeta potential instrument (ZEN3700, Malvern Instruments Co., Ltd., Worcestershire, UK). Microbial culture was carried out in a constant temperature and humidity incubator (CHS-100CL, Shanghai Yiheng Scientific Instrument Co., Ltd., Shanghai, China). Microbial morphology was observed by an optical microscope (SZ40, Olympus Co., Ltd., Phoenix, Curepipe, Mauritius).

The content of mycotoxins was measured by a gas chromatograph–mass spectrometer (GC-MS, TSQ8000, ThermoFisher Scientific Co., Ltd., Waltham, MA, USA). The detection procedure was as follows: two microliters of samples were injected and performed in splitless mode at 300 °C. The temperature was firstly kept at 150 °C for 1 min, then increased to 280 °C at a rate of 10 °C/min, then further ramped to 310 °C at a rate of 30 °C/min, and finally kept at 310 °C for 5 min. Electronic-impact energy was set to −70 eV. The chromatographic column was ZB-WAX (60 m × 0.25 mm × 0.25 µm). The carrying gas was helium. Trimethylsiliconether derivatives of mycotoxins were identified by the following fragment ion (M/Z). The quantitative ions of mirex, DON, 3ADON, and 15ADON were 271.9, 295.2, 377.2, and 392.2, respectively.

Ergosterol content was measured by a high-performance liquid chromatography (HPLC, Agilent 1260, Agilent Technologies Co., Ltd., of Shanghai, Shanghai, China). The detection procedure was as follows: the chromatographic column was Agilent Eclipse XDB-C18, the column temperature was 30 °C, the injection volume was 20 μL, the flow rate was 1.0 mL/min, the mobile phase was methanol, and the detection wavelength was 282 nm.

### 2.3. Isolation and Identification of Fusarium from Barley

#### 2.3.1. Fusarium Isolation and ITS Identification

A proper amount of barley grains infected with *Fusarium* head blight were crushed and washed in sterile saline for 5 min to obtain a suspension containing microorganisms from barley. The suspension was coated on PDA plates and cultured at 28 °C for 48–96 h. Then, the single colony mycelia with obvious filamentous fungal morphology were selected for purification culture. Finally, the obtained strains were stored at 4 °C.

The filamentous fungi screened were extracted with plant genome extraction kit to obtain the whole genome. Then, the whole genome was sent to Tianlin Biotechnology Co., Ltd., (Shanghai, China) for ITS sequencing. Blast comparison was performed on NCBI and the phylogenetic tree was drawn by MEGA software (MEGA6.0).

#### 2.3.2. Identification of Toxins Produced by Fusarium

A total 4 mL of *Fusarium* spore (10^6^ CFU/mL) was added to 80 g of sterile barley medium. After inoculation, the barley culture medium was placed at 28 °C for 7 days (95% humidity) to produce toxin. After the toxin was produced, the medium was collected (freeze-dried) and crushed to determine the mycotoxin [26]. All experiments were carried out three times.

#### 2.3.3. Morphological Identification

*Fusarium* F-4 was cultured in the PDA medium for 4 days to observe its morphology.

### 2.4. Preparation and Screening of Different Essential Oil Primary Emulsions

#### 2.4.1. Preparation of Primary Emulsion

Emulsifier solution: 1% lecithin was dissolved in 10 mmol/L acetate buffer (pH = 3.0) and stirred in the ice bath to dissolve. Oil phase: essential oil and medium-chain triglyceride (MCT) were used as oil phase. Preparation of primary emulsion: 92 g emulsifier solution was mixed with 8 g oil phase and homogenized for 2 min with a handheld homogenizer. Then, the emulsion with good initial homogenization continued homogeneity of 1 min at 100 MPa at a rate of 9 L/h.

#### 2.4.2. Antifungal Properties of Different Essential Oils

The inhibition zone method was as follows: 100 μL spore solution with a concentration of 10^5^ CFU/mL was coated on the PDA plate. Then, a 6 mm-diameter hole punch was used to drill a hole in the center of the plate. Next, 10 μL of essential oil were added to the holes, and the culture medium was cultured at 27 °C for 4 days. Finally, the diameters of inhibition zones were measured by a vernier caliper. All experiments were carried out three times.

According to the method of Kalagatur et al. [27], the minimum inhibitory concentration (MIC) and minimum bactericidal concentration (MBC) of essential oil against *Fusarium* were determined by microporous dilution method. A total 10 μL spore solution (10^6^ CFU/mL) was added to a 96-well plate and mixed with different concentrations of essential oils. Then, the volume was added to 100 μL with potato medium (PDA) of liquid. The wells without essential oils were used as blank control. The final concentration of essential oil was 0, 125, 250, 500, 1000, 2000, and 4000 μg/mL. After culturing at 27 °C for 3 days, the minimum essential oil concentration for the growth of no visible fungi was MIC. Then, after 7 days of continuous culture, 50 μL medium was taken from 96-well plates and coated on PDA plates. After culturing at 27 °C for 3 days, the minimum essential oil concentration for the growth of no visible fungi was MBC. Three parallel tests should be performed for each concentration.

#### 2.4.3. Effect of Different Primary Emulsions on Germinability of Barley

Two pieces of 9 cm-diameter filter paper were placed on the bottom of a petri dish, and different concentrations of primary emulsions were added to the filter paper. Then, 100 grains of barley were added to the petri dish and incubated at 20 °C for 2–3 days to observe the germination number of barley. Germinability = the number of germinated barley/the number of total barley. Tween 80 solution was used as a blank control. All experiments were carried out three times.

### 2.5. Preparation and Performance Study of Chitosan-Based Secondary Emulsion

#### 2.5.1. Preparation of *Litsea cubeba* Essential Oil Secondary Emulsion

Preparation of chitosan solution: 1% chitosan was dissolved in 10 mmol/L acetate buffer (pH = 3.0) and stirred in an ice bath to dissolve. Preparation of secondary emulsion [28]: chitosan solutions with different concentrations were mixed with the primary emulsion by 1:1, and then ultrasonic was performed at a power of 40 kHz and 300 W for 30 s. The experiment was repeated 3 times, and then stood at room temperature for 24 h.

#### 2.5.2. Study on the Physical Stability of Chitosan-Based Secondary Emulsion

The physical stability of chitosan-based secondary emulsion with different concentrations was characterized by average particle size and zeta potential [28]. Subsequently, the pH stability and storage stability were further investigated by changing the pH and storage time [28]. All experiments were carried out three times.

#### 2.5.3. Study on Antifungal Properties of Chitosan-Based Secondary Emulsion

The emulsion was diluted to different concentrations with acetic acid buffer and 100 μL diluted emulsion was coated on the PDA plate. Then, fresh *Fusarium graminearum* cake with a diameter of 6 mm was inoculated on the medium. After incubation at 27 °C for 4 days, the diameter of mycelia was measured [29]. Mycelial growth inhibition (MGI) (%) = (blank mycelium diameter − sample mycelium diameter)/(blank mycelium diameter − cake diameter) × 100%. The experiments were carried out using the same concentrations (0.1%) of emulsions and chitosan solution. All experiments were carried out three times.

#### 2.5.4. Study on Germinability of Chitosan-Based Secondary Emulsion

The experimental procedure was the same as that described in Section 2.4.3. Germinability experiments were carried out through using the same concentration (1 mg/g and 5 mg/g) of chitosan-based secondary emulsion and primary emulsion. All experiments were carried out three times.

### 2.6. Application of Secondary Emulsion in Malting

The malting process was as follows: 200 g of barley infected with *Fusarium* head blight was used as a typical example. Soaking process: barley was soaked in water for 6 h, dried for 8 h, then soaked for 4 h, dried for 6 h, and finally soaked for 3 h (the soaking temperature was 13 °C, the humidity was 95%, and the soaking degree was controlled at 43–45%). Germination: the barley was cultured at 14 °C for 1 day, 14.5 °C for 1 day, and 15 °C for 3 days, respectively (barley was stirred twice a day and watered once a day). Baking: the barley was baked at 57 °C for 7 h, 59 °C for 2 h, 61 °C for 2 h, 63 °C for 2 h, 65 °C for 2 h, 73 °C for 2 h, 77 °C for 1 h, and 86 °C for 3 h.

To evaluate the effect of the addition amount of secondary emulsion on the DON content, ergosterol content, germination rate, and other indicators of finished malt after the end of the barley production, the amount of secondary emulsion in the third stage of soaking process was changed. All experiments were carried out three times.

### 2.7. Statistical Analysis

Data were collected using Microsoft Excel 2013 (Microsoft Corporation, Redmond, WA, USA), the mean value and standard deviation of data were calculated by SPSS (SPSS 24.0, International Business Machines Corporation, Armonk, NY, USA), and these data were imported into Origin (OriginPro8.0, Originlab Corporation, Northampton, MA, USA) to draw the graphics.

## 3. Results and Discussion

### 3.1. Isolation and Identification of Fusarium

#### 3.1.1. Identification of Fusarium by ITS

**Figure 1:**
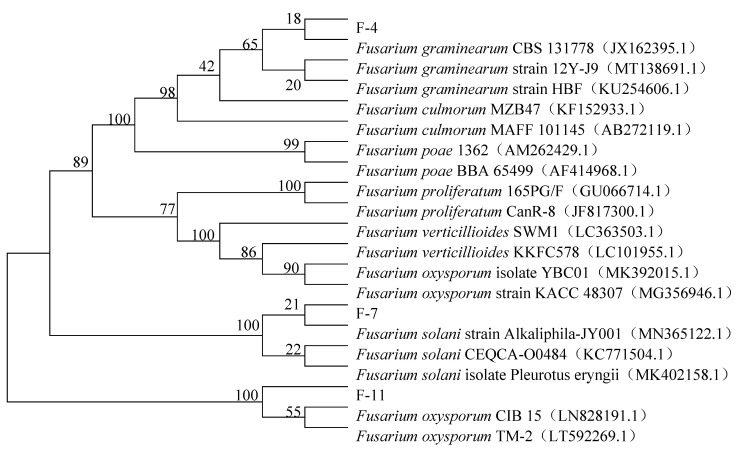
ITS sequence phylogenetic tree of three *Fusarium* strains.

Thirteen strains of filamentous fungi from barley infected with *Fusarium* head blight were obtained by plate coating and further purification. The obtained fungi were sequenced by ITS and compared by BLAST on NCBI. Three strains of *Fusarium* were identified according to the comparison results, and phylogenetic trees were constructed by sequencing and comparing results of the three strains. As shown in Figure 1, *Fusarium* F-4 had closer homology with *F. graminearum CBS 131778*, *Fusarium* F-7 had closer homology with *F. solanum ALKaliphila-JY001*, and *Fusarium* F-11 had closer homology with *F. oxysporum CIB 15 and TM-2*.

#### 3.1.2. Analysis of Toxin Produced by Fusarium

The toxin content of barley infected with *Fusarium* head blight was detected by GC-MS [26]. Only DON and 15ADON were detected in the samples, with the content of 0.43 and 0.12 mg/kg, respectively, and 3ADON was not detected, indicating that the pathogen was *F. graminearum* of 15ADON chemical type [30]. The three *Fusarium* strains isolated earlier were inoculated into barley culture medium for toxin producing experiment. As shown in Table 1, it was found that DON and 15ADON could be detected only in barley culture medium inoculated with *Fusarium* F-4, and the yields were 23.61 and 215.44 mg/kg, respectively, indicating that DON and its derivatives were produced by *Fusarium* F-4.

#### 3.1.3. Morphology of Fusarium F-4

**Figure 2:**
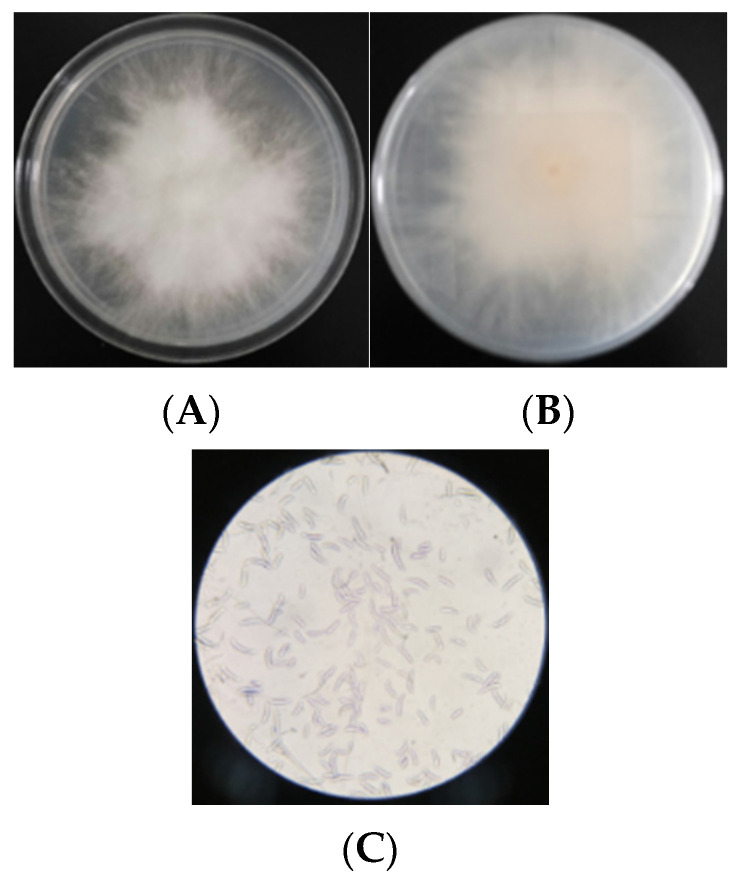
Morphological photograph of isolated *Fusarium* F-4. (**A**) is the image of the front of the petri dish, (**B**) is the image of the back of the petri dish, and (**C**) is the microscopic image of spores.

As shown in Figure 2A,B, aerial mycelia of *Fusarium F-4* were dense with white cotton flocs, and there was a small amount of orange pigment deposition in the central position on the back of the plate in the later stage of culture. Spores were collected by continuous culture in MBA plate and CMC medium and their morphology was observed. As shown in Figure 2C, the conidia, with 2–4 transverse septa, were sickle shaped, and their lengths and widths were 8.7–29.4 μm and 2.2–3.2 μm, respectively.

### 3.2. Screening and Performance Analysis of Primary Emulsion

#### 3.2.1. Inhibitory Effect of Different Essential Oils on Fusarium

DON in malt is mainly produced by *Fusarium* during malting. In general, the inhibition of essential oil on mycotoxin production is mainly achieved by inhibiting the growth of fungi. Three isolated strains of *Fusarium* were selected as test strains to evaluate the antifungal activity of essential oils. For antifungal experiments, seven kinds of plant essential oils were selected, including clove oil, oregano oil, cinnamon oil, citronella oil, *Litsea cubeba* oil, fennel oil, and patchouli oil.

The results of inhibition zone method were shown in Table 2. The results showed that clove oil, oregano oil, cinnamon oil, *Litsea cubeba* oil, and citronella oil had good inhibitory effects on the three strains of *Fusarium*, and fennel oil and patchouli oil had almost no inhibitory effect. MIC and MBC of five essential oils with good inhibitory effect were determined by microporous dilution method. In Table 3, citronella oil has a very poor antifungal effect (both MIC and MBC were 4000 μg/mL), while the antifungal effect of cinnamon oil was best (both MIC and MBC were within 250 ug/mL). In addition, the MIC and MBC of clove oil, oregano oil, and *Litsea cubeba* oil were relatively small, which were consistent with the inhibition zone method. Therefore, cinnamon oil, clove oil, oregano oil, and *Litsea cubeba* oil were used for subsequent experiments.

#### 3.2.2. Effect of Different Primary Emulsions on Germinability of Barley

**Figure 3:**
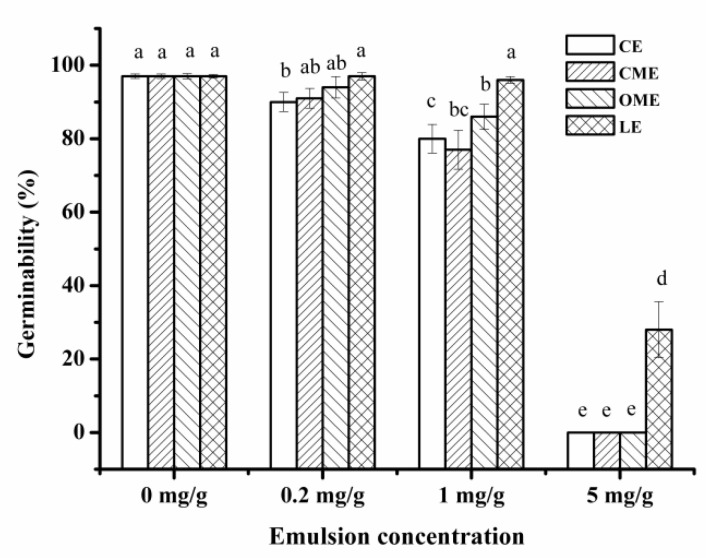
The effect of four kinds of primary emulsions on the germinability of barley. CE is clove oil emulsion, CME is cinnamon oil emulsion, OME is oregano oil emulsion, and LE is *Litsea cubeba* oil emulsion.

Germinability is one of the important indexes to evaluate the regularity of barley germination. Cinnamon oil, clove oil, oregano oil, and *Litsea cubeba* oil were used as raw materials to prepare primary emulsions and the primary emulsion suitable for malting was selected. As shown in Figure 3, all emulsions had a certain inhibitory effect on the germinability of barley, and the inhibitory effect increased with the increase in concentration. Moreover, the germinability of barley treated with *Litsea cubeba* oil was significantly higher than that of other groups. The germinability of barley treated with *Litsea cubeba* oil emulsion was 96% when the emulsion concentration was 1 mg/g, and the germinability of barley treated with *Litsea cubeba* oil emulsion was still 28% when the emulsion concentration reached 5 mg/g. As for other essential oils, the germinability of barley was 0% when the emulsion concentration reached 5 mg/g. So, *Litsea cubeba* oil had the least inhibitory effect on barley germination.

### 3.3. Effect of Chitosan on the Performance of Secondary Emulsion

#### 3.3.1. Effect of Chitosan on Physical Stability of Secondary Emulsion

**Figure 4:**
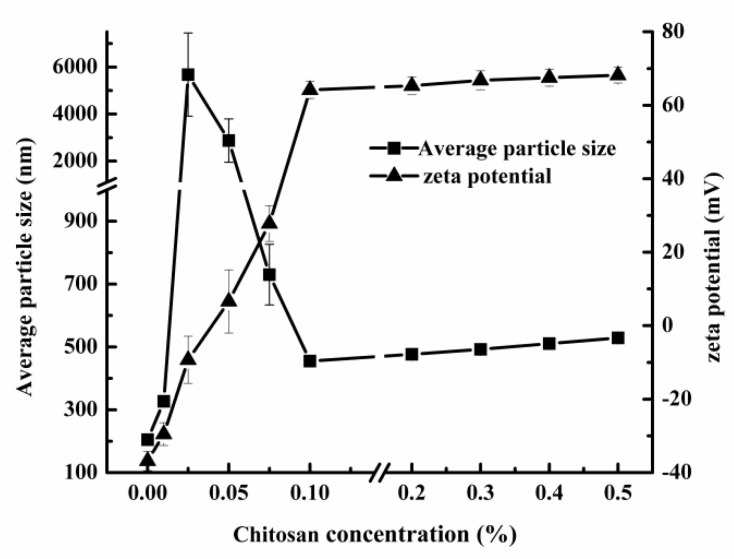
Effect of chitosan concentration on average particle size and zeta potential of secondary emulsion.

The influence of different concentrations of chitosan on the stability of secondary emulsion was studied. In Figure 4, the zeta potential gradually changed from −36.8 to 64.2 mV with the increase in chitosan concentration when the concentration of chitosan was 0−0.1%, indicating that chitosan was successfully used as the wall material of emulsion [28]. For a more detailed description, we found that the average particle size of secondary emulsion increased greatly when zeta potential tended to be 0 mV and the average particle size decreased when zeta potential continued to increase, indicating that the stability of emulsion also decreased first and then increased with the increase of chitosan [28]. When the concentration of chitosan was greater than 0.1%, the zeta potential and average particle size increased slightly with the increase in concentration. In addition, the presence of excess chitosan in the solution promoted the deposition of a small amount of chitosan with the increase in intermolecular forces, resulting in a slight increase in zeta potential and average particle size. In order to reduce the introduction of other substances in the emulsion except essential oils, 0.1% chitosan was finally selected for the additive amount of the secondary emulsion.

**Figure 5:**
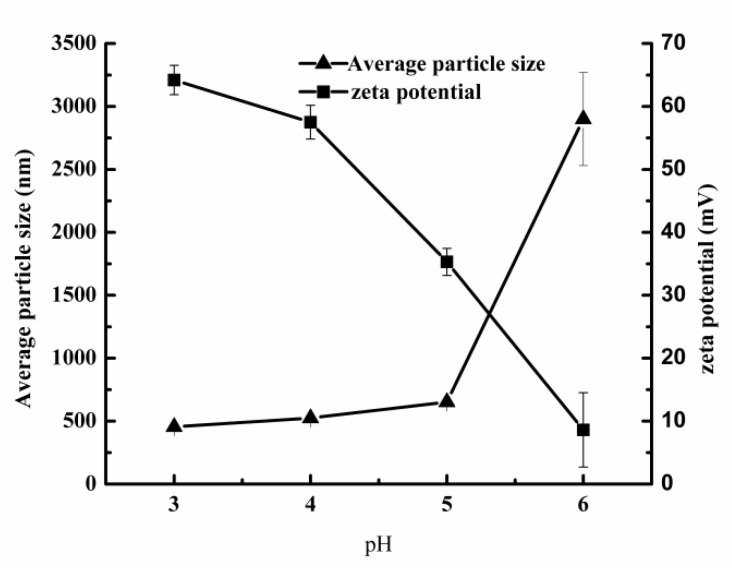
Effect of pH on average particle size and zeta potential of secondary emulsion.

The pH stability of the secondary emulsion was studied by measuring the average particle size and zeta potential of the secondary emulsion at different pH values. As shown in Figure 5, with the increase of pH from 3.0 to 5.0, the average particle size of the secondary emulsion increased slightly, and the zeta potential decreased obviously. The average particle size and zeta potential of the secondary emulsion at pH = 5.0 were 651.7 nm and 35.3 mV, respectively, indicating that secondary emulsion of pH = 5.0 still had good stability [28]. When the pH of secondary emulsion increased to 6.0, the particle size increased to 2.9 μm and the zeta potential dropped to 8.6 mV, indicating that the stability of secondary emulsion became poor [28]. Therefore, the prepared secondary emulsion had better stability in the pH range of 3.0–5.0.

**Figure 6:**
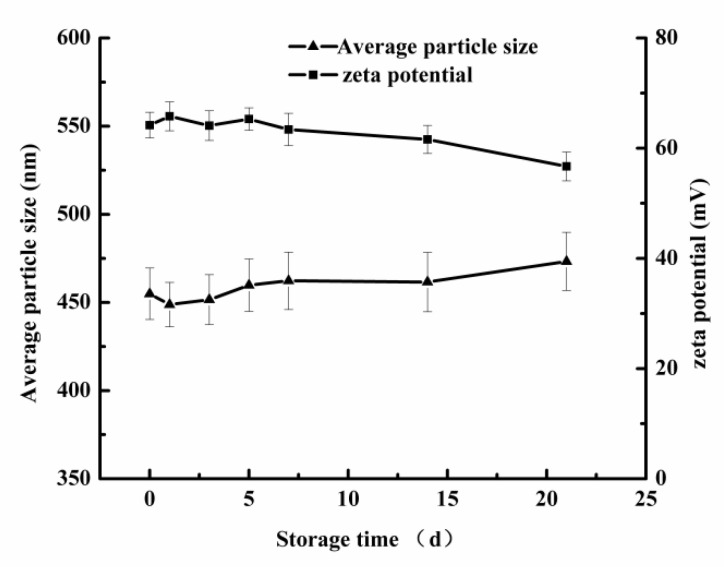
The particle size and zeta potential of secondary emulsion after 21 days storage.

The pH value with the smallest particle size was selected for storage experiment. The storage stability of the secondary emulsion (pH = 3) within 21 days at room temperature was studied. As shown in Figure 6, with the increase in storage days, the average particle size of the emulsion increased slightly, and the zeta potential decreased slightly. On the 21st day, the zeta potential and average particle size of the emulsion were 56.7 mV and 473.3 nm, respectively, which showed no difference from the initial state. Therefore, the prepared secondary emulsion had better storage ability.

#### 3.3.2. Effect of Chitosan on Antifungal Performance of Secondary Emulsion

**Figure 7:**
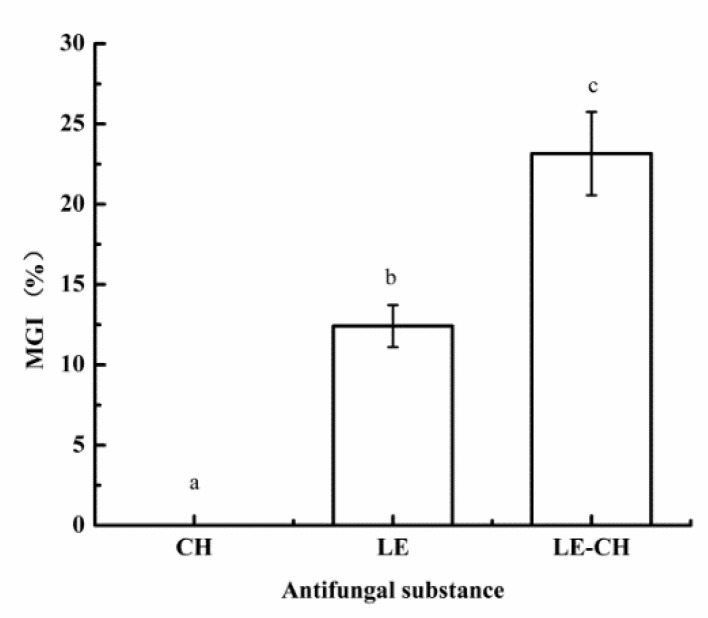
Effect of chitosan on the inhibition of *Fusarium graminis* by *Litsea cubeba* oil emulsion. CH represents chitosan solution, LE represents *Litsea cubeba* oil primary emulsion, and LE-CH represents *Litsea cubeba* oil secondary emulsion.

The effect of chitosan on the antifungal effect of the emulsion was evaluated by using the same concentration (0.1%) of emulsions and chitosan solution. As shown in Figure 7, compared with the primary emulsion, the MGI of the secondary emulsion with chitosan increased from 12.4% to 23.1% at the same concentration of essential oil, indicating that the addition of chitosan can improve the antifungal effect of essential oil emulsion. It is worth noting that 0.1% chitosan did not inhibit the mycelia growth of *Fusarium graminis*, which further illustrated that the reason for the enhanced effect of chitosan was that it can act as a wall material to delay the release of essential oils [27].

#### 3.3.3. Effect of Chitosan on Germinability of Barley

**Figure 8:**
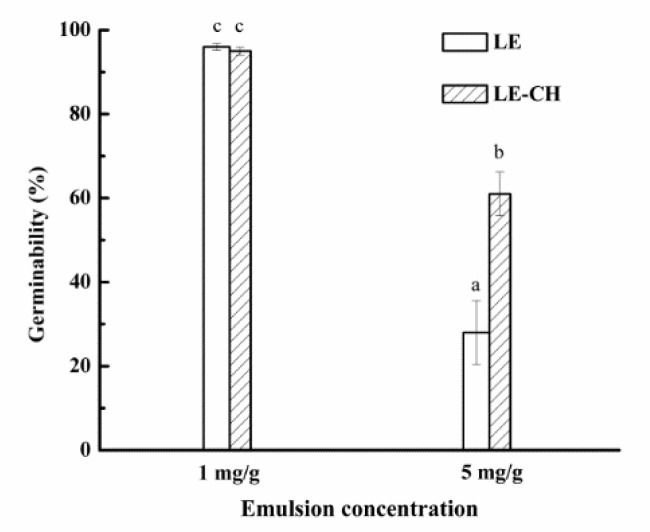
Effect of chitosan on germinability of *Litsea cubeba* oil emulsion. LE represents *Litsea cubeba* essential oil primary emulsion and LE-CH represents *Litsea cubeba* essential oil secondary emulsion.

Germinability experiments were carried out through using the same concentration (1 and 5 mg/g) of chitosan-based secondary emulsion and primary emulsion. The results are shown in Figure 8. The germinability of barley increased from 28.0% to 61.0% after adding chitosan when the concentration of essential oil was high (5 mg/g), and the germinability of barley had no inhibitory effect after adding chitosan when the concentration of essential oil was low (1 mg/g), indicating that the inhibitory effect of *Litsea cubeba* oil on germinability of barley was decreased by 0.1% chitosan. The reason may be that the primary emulsion was more likely to break in the water shortage environment, leading to a large amount of essential oil volatilization, and chitosan as a wall material delayed the release of essential oil [27].

### 3.4. Effect of Secondary Emulsion on Malting

#### 3.4.1. Effect of Secondary Emulsion on Germination Rate and DON Accumulation

**Figure 9:**
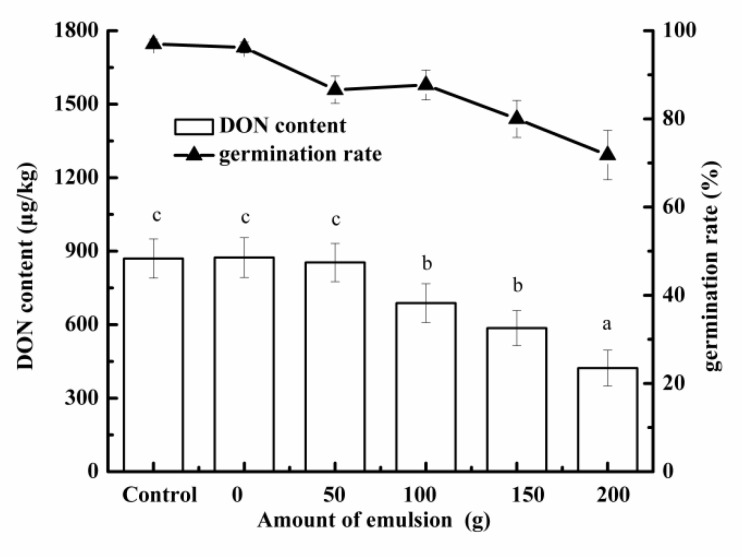
Effect of adding secondary emulsion during third soaking on germination rate of barley and DON content in malt.

As shown in Figure 9, with the increase in emulsion content in the third soaking stage, the germination rate of barley decreased gradually, and the DON content of finished malt decreased significantly. For a more detailed description, when the supplemental level was 100 g, the germination rate of barley was 87.7% and the DON concentration of finished malt was reduced to 690 μg/kg, which was 20.9% lower than that of the control; when the dosage was 200 g, the germination rate of barley was 71.8% and the DON concentration of finished malt was reduced to 420 μg/kg, which was reduced by 51.4% compared with the control. It can be seen that the dosage of secondary emulsion applied to malting needed to be balanced.

#### 3.4.2. Effect of Secondary Emulsion on Ergosterol Accumulation during Malting

**Figure 10:**
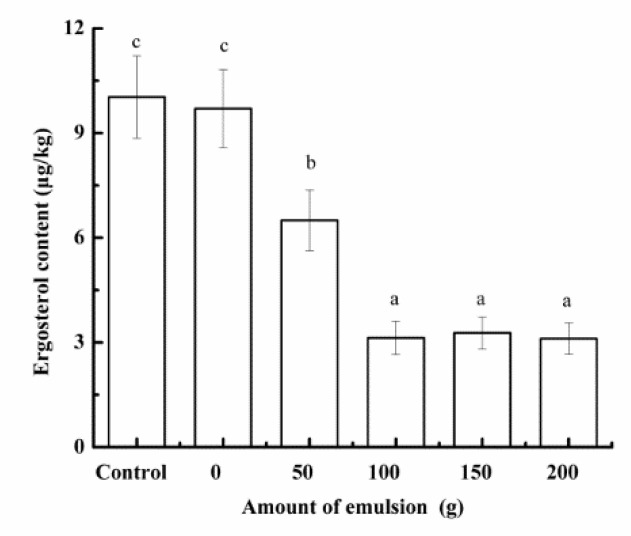
Effect of adding secondary emulsion during third soaking on ergosterol content in malt.

The ergosterol content was tested to evaluate the effect of secondary emulsion on the fungal biomass [28]. In Figure 10, when the addition amount of the emulsion was less than 100 g, the ergosterol content gradually decreased with the increase in the addition amount of the emulsion. This showed that the secondary emulsion can effectively inhibit the growth of fungi. However, when the addition amount of the emulsion was greater than 100 g, the inhibitory effect of the emulsion on the fungal biomass did not increase significantly. The growth of some fungus resistant to *Litsea cubeba* oil in barley may be the reason why it did not decrease in the later period.

#### 3.4.3. Effect of Secondary Emulsion Treatment on Final Malt Quality

The malts prepared by three different treatments were tested for some conventional malt indexes; the results are shown in Table 4. Compared with the control group (A), malt (B) prepared with the addition of primary emulsion had lower value of extractum and Kolbach, longer saccharification time, and significantly lower filtering rate and higher chromaticity. The results showed that the addition of primary emulsion had a great influence on the quality of malt and the feasibility of practical application was not high. For the malt (C) processed by secondary emulsion, almost all indexes of malt were improved compared with that prepared by primary emulsion, which showed that chitosan could reduce the influence of primary emulsion on the quality of malt. The reason may be that the slow-release effect of chitosan improved the environment of malt germination and reduced the interference of essential oil to malt germination [27].

## 4. Conclusions

In our work, the primary emulsion of *Litsea cubeba* oil with strong antifungal properties and little effect on germinability of barley were screened. Then, the effect of chitosan on the properties of *Litsea cubeba* essential oil primary emulsion was studied. Fortunately, the addition of chitosan can improve the physical stability and antifungal ability of the emulsion and reduce the effect of the emulsion on barley germination. Finally, chitosan-based *Litsea cubeba* essential oil emulsion was applied to malting process. The results showed that the addition of primary emulsion had a great influence on the quality of malt, but the effect can be reduced by adding chitosan into the emulsion. The addition of chitosan-based secondary emulsion not only inhibited the accumulation of DON during malting, but also enhanced the quality of malt. This research can promote the application of EOs emulsion in malt industry to produce safe and high-quality malt. In addition, it was found that the application of EOs emulsion in the malting process could affect barley germination; however, the possible mechanism could not be explained at present. In the future, we will continue to study this in order to better apply the emulsion to the beer industry.

## Figures and Tables

**Table 1:** Determination results of DON and its derivatives produced by *Fusarium* strains.

Strains	Toxin Content (mg/kg)		
	DON	3ADON	15ADON
F-4	23.61 ± 0.52	-	215.44 ± 0.95
F-7	-	-	-
F-11	-	-	-

Note: “-” indicates not detected.

**Table 2:** Inhibition zone diameter of seven essential oils against *Fusarium* strains.

Essential Oil	Diameter of Inhibition Zone (mm)		
	*Fusarium solanum*	*Fusarium oxysporum*	*Fusarium graminis*
Blank	0	0	0
Clove oil	35.3 ± 1.2	33.3 ± 1.4	44.9 ± 2.0
Oregano oil	29.0 ± 1.1	33.6 ± 1.4	44.2 ± 2.4
Cinnamon oil	42.2 ± 3.2	32.7 ± 2.3	58.3 ± 0.6
Citronella oil	16.1 ± 1.2	18.3 ± 0.6	20.3 ± 1.3
*Litsea cubeba* oil	30.6 ± 0.7	36.1 ± 0.9	30.1 ± 1.0
Fennel oil	0	0	0
Patchouli oil	0	0	0

**Table 3:** MIC and MBC of five plant essential oils against three *Fusarium* strains.

Essential Oil	Essential Oil Content (μg/L)					
	*Fusarium solanum*		*Fusarium oxysporum*		*Fusarium graminis*	
	MIC	MBC	MIC	MBC	MIC	MBC
Clove oil	500	1000	500	1000	500	1000
Oregano oil	1000	2000	1000	2000	1000	1000
Cinnamon oil	250	250	125	125	250	250
*Litsea cubeba* oil	1000	2000	500	500	2000	2000
Citronella oil	>4000	>4000	>4000	>4000	>4000	>4000

**Table 4:** Effect of essential oil emulsion on malt index.

Index	A	B	C
Moisture (%)	4.89 ± 0.02	4.64 ± 0.02	4.81 ± 0.03
Extractum (%)	79.60 ± 0.80	72.20 ± 0.40	76.20 ± 0.50
Saccharification time (min)	10	20	10
Filtering rate (min/100 mL)	13.42 ± 1.52	46.75 ± 2.20	28.25 ± 1.25
pH	6.23 ± 0.22	5.93 ± 0.01	6.19 ± 0.05
Chromaticity (EBC)	6.13 ± 1.25	14.13 ± 1.02	7.00 ± 0.08
Turbidity (EBC)	2.36 ± 0.36	4.83 ± 0.75	3.14 ± 0.68
α-amino nitrogen (mg/100 g)	154.05 ± 1.20	152.16 ± 1.15	158.43 ± 0.04
Kolbach index	38.58 ± 1.02	32.73 ± 1.12	37.59 ± 0.52

Note: A is the control group, B is the malt prepared with the addition of primary emulsion, and C is the malt processed by secondary emulsion.

## Data Availability

Not applicable.
